# High-frequency visual stimulation can increase medial temporal lobe ripple oscillation density

**DOI:** 10.1038/s43856-026-01846-6

**Published:** 2026-08-10

**Authors:** Julian Keil, Victor Hernandez-Urbina, Liam Doherty, Hamed Bahmani, Martin Holtkamp, David Steinbart, Alexandra-Dikorey Diall, Jonas M. Hebel, Christian Brandt, Thomas Cloppenborg, Anna Doll, Markus Müschenich

**Affiliations:** 1 University of Potsdam, Department of Psychology, Potsdam, Germany; 2 Nuuron GmbH, Berlin, Germany; 3 Charité – Universitätsmedizin Berlin, Clinical Neurotechnology Laboratory, Department of Psychiatry and Psychotherapy, Berlin, Germany; 4 International Center for Primate Brain Research (ICPBR), Center for Excellence in Brain Science and Intelligence Technology, Chinese Academy of Sciences, Shanghai, China; 5 Epilepsy-Center Berlin-Brandenburg, Institute for Diagnostics of Epilepsy, Berlin, Germany; 6 Charité – Universitätsmedizin Berlin, Department of Neurology, Berlin, Germany; 7 Department of Epileptology, Krankenhaus Mara, Epilepsy-Center Bethel, Bielefeld University, Medical School OWL, Bielefeld, Germany

**Keywords:** Hippocampus, Target validation

## Abstract

**Background:**

Flickering visual stimulation can evoke neural oscillations, which can influence ongoing brain activity. Electrophysiological recordings of neural oscillations in the ripple band (80–180 Hz) show that these high-frequency oscillations occur in the neocortex and the hippocampus, that they phase-synchronize across long distances, and that ripple oscillations in the neocortex often precede those in the hippocampus during wakefulness. It is therefore possible that the neocortical ripple oscillations propagate beyond sensory areas to the hippocampus, inducing ripple oscillations.

**Methods:**

To test the hypothesis that neocortical ripple oscillations induced by visual stimulation induce hippocampal ripple oscillations, we conduct an exploratory experiment (*N* = 8) in humans, using ultra-high frequency visual stimulation to induce ripple oscillations recorded through electrodes implanted in or near the hippocampus. Although hippocampal ripple oscillations, so-called sharp-wave-ripples, mostly occur during quiet rest or slow-wave sleep, we aim to increase their abundance using visual stimulation during wakefulness in this exploratory study. We hypothesize that ultra-high frequency visual stimulation increases the number of sharp-wave-ripples relative to an eyes-open resting-state baseline.

**Results:**

In this exploratory and preliminary study, we observe significantly more sharp-wave-ripples per second during periods of stimulation compared to a resting-state baseline before and after the stimulation.

**Conclusions:**

The increased number of sharp-wave ripples during stimulation suggests that ultra-high-frequency visual stimulation can be used as a safe noninvasive tool to influence sharp-wave ripples, which offers the potential to improve memory.

**Figure:**
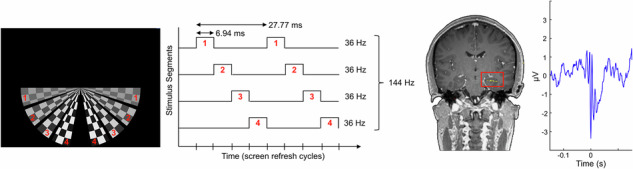

## Plain language summary

This exploratory study examines a noninvasive way to influence brain activity linked to memory. Sharp-wave ripples (SWRs) in the hippocampus are important for forming and recalling memories, but they are reduced in aging and Alzheimer’s Disease. The experiment tests whether very fast flickering light, called ultra-high frequency visual stimulation (UHV-stimulation), can increase the number of SWRs. Eight epilepsy patients with implanted electrodes receive UHV-stimulation while their brain activity is recorded. The results suggest that UHV-stimulation increases the number of SWRs compared to a resting baseline. Other properties of SWRs do not change, and participants report no negative effects. These preliminary results suggest that UHV-stimulation might support memory in the future.

## Abstract (web summary)

Keil et al. use visual stimulation at ultra-high frequencies in an exploratory study with 8 participants to test whether neocortical ripple oscillations can induce hippocampal sharp-wave ripples recorded by implanted electrodes. Ultra-high frequency visual stimulation significantly increases the rate of hippocampal sharp-wave ripples compared with an eyes-open resting-state baseline, suggesting a safe, non-invasive approach to modulating ripple activity with potential memory-related applications, although validation in larger cohorts is required.

## Introduction

A long line of human and animal research has established that sharp-wave ripples (SWRs or SPW-R) play a central role in memory consolidation and recall^1–3^. SWRs are short episodes (~100 ms) of synchronous high-frequency (~80–180 Hz in humans) oscillations, which are accompanied by a large-amplitude sharp wave^4^. They were first described in the hippocampus^1^, and subsequent research has also demonstrated ripple oscillations in the neocortex^5^. Ripple oscillations synchronize local neural networks and distant areas of the brain to facilitate information exchange^6^, making them a core neural mechanism of episodic memory formation, consolidation, and retrieval^2,7^.

The number of SWRs after memory encoding is strongly correlated with the number of recalled memory items^8^. Similarly, SWRs locked to spindle activity during sleep are correlated with memory reactivation^9^. In causal experiments in mice, increasing the number or duration of SWRs positively influenced learning and memory^10,11^, while impairing SWRs inhibited memory recall^12,13^. SWRs have also been implicated in psychiatric conditions affecting memory such as Alzheimer’s Disease (AD)^14,15^. The number, power, and frequency of SWRs are reduced in mouse models of AD^16^, which could explain the observed cognitive impairment^17,18^. Overall, correlational evidence from humans and causal evidence from mice suggest that increasing the number of SWRs may improve memory, which could pave the way for treatments of cognitive impairment in dementia or due to normal aging.

Inducing SWRs in vitro and in vivo is possible with various invasive stimulation approaches^19–21^. However, these are not suitable for use in humans (e.g., optogenetic manipulations) or for broad application as an early intervention or prevention measure (e.g., invasive electrical stimulation). Invasive electrical stimulation of the Fornix can have cognitive benefits in human AD patients, but the underlying mechanism of action is still unclear^22^. Noninvasive stimulation approaches (e.g., repetitive transcranial magnetic stimulation, rTMS) or visual gamma band stimulation also promise immediate and long-term improved cognition in AD patients, but the underlying mechanisms of action are similarly unclear^23–25^. Thus, there is a gap between applications that are safe and easy to use in humans and stimulation protocols targeted directly at the neural mechanisms of memory.

To overcome this challenge, we propose a noninvasive stimulation approach comprising spatio-temporally coordinated ultra-high frequency visual stimulation (UHV-stimulation) using intensity-modulated light impulses. This stimulation approach uses visual stimulation frequencies in the range of neocortical ripples and hippocampal SWRs (80-180 Hz) to induce neocortical oscillations in the ripple frequency range^26,27^. Neocortical ripple oscillations precede hippocampal SWRs^6^. Thus, evoking neocortical ripple oscillations using UHV-stimulation could, plausibly, induce hippocampal SWRs. Following this line of argumentation, we hypothesize that UHV-stimulation would increase the number of SWRs compared to a resting state baseline. We test this hypothesis in an exploratory experiment with precise recordings from the medial temporal lobe in humans using invasive electrodes. In line with this hypothesis, we find an increased number of SWRs per second during high-frequency visual stimulation compared to eyes-open resting state recording with constant light stimulation. This preliminary finding opens the door to exploring UHV-stimulation as a tool to improve memory.

## Methods

In the current exploratory experiment, we tested the effects of UHV-stimulation on neural activity in humans recorded from implanted electrodes while participants were exposed to different versions of the UHV stimulation and during resting-state baseline. Processed data and scripts to reproduce the analyses are available as open data in a Center for Open Science repository (10.17605/OSF.IO/YCK7T).

### UHV-stimulation

Repetitive visual stimulation can lead to steady-state visual evoked potentials (SSVEP) in visual cortical areas^28,29^. SSVEP stimulation can also be used to stimulate multiple locations in the visual field simultaneously^30,31^. While SSVEP at high frequencies has been reported^32,33^, the peak latency of retinal photoreceptors (approximately 10 ms^34,35^) could explain the difficulty of evoking responses above 100 Hz.

**Fig. 1:**
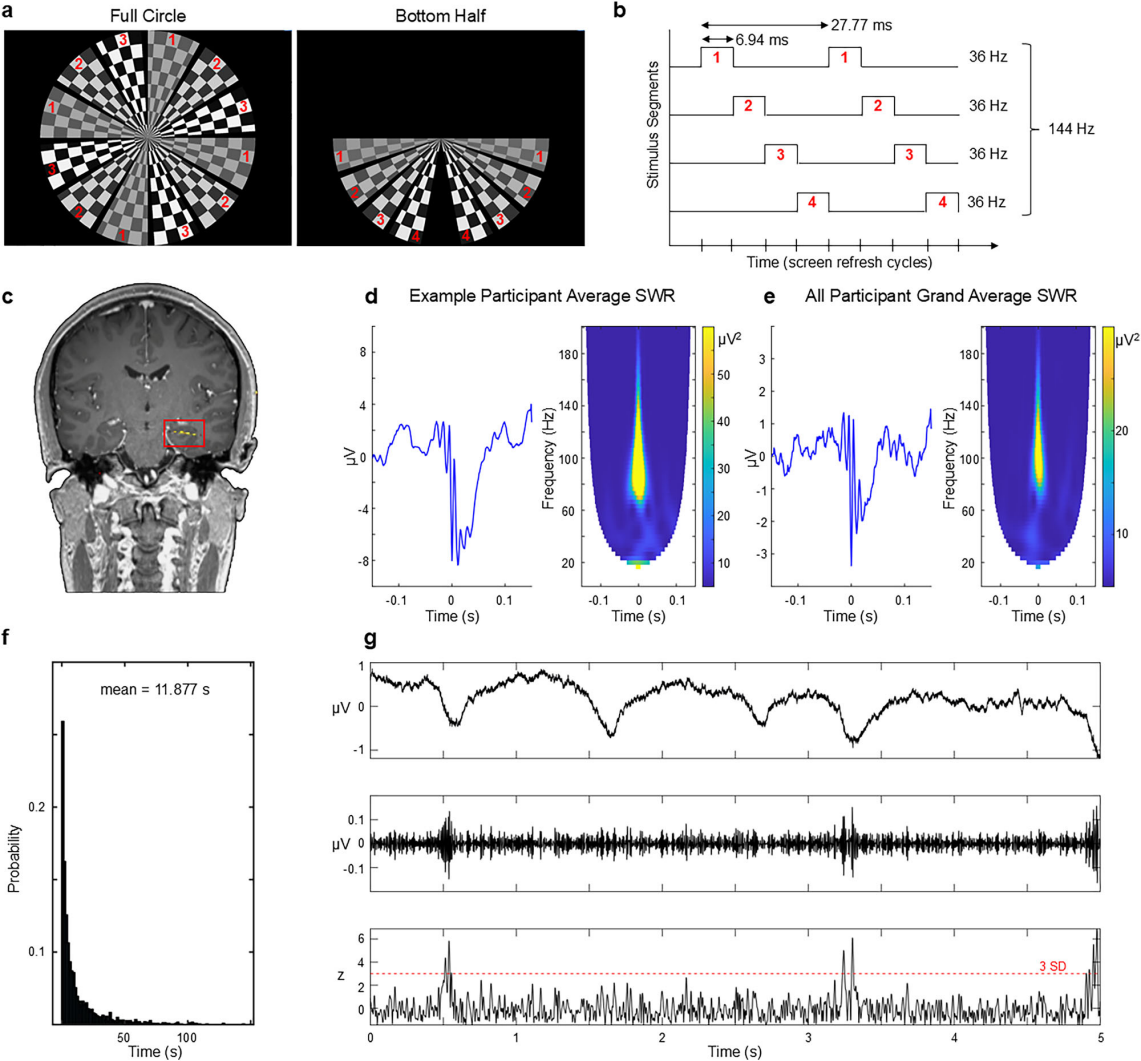
Summary of the principle of UHV-stimulation and SWR properties. **a** Illustration of the two different UHV-stimulation conditions. The full circle (FC) stimulus comprised twelve 30° sections, of which four were displayed simultaneously before the clockwise neighboring sections were displayed. The bottom half (BH) stimulus comprised eight 20° sections, of which two were displayed simultaneously before the lower neighboring sections were displayed. Red numbers indicate the order of stimulation and were not visible during the experiment. **b** Illustration of spatio-temporal stimulation in the bottom half stimulation condition. The stimulation comprises four segments. Each segment is displayed for one screen refresh cycle. During the first cycle, the first segment is displayed, the second segment is then displayed during the second cycle, the third during the third cycle and the fourth during the fourth cycle before the stimulation starts again with the first segment. Each segment therefore is displayed at a frequency of the screen refresh rate divided by the number of segments. **c** Illustration of the intracranial electrode placement in the medial temporal lobe of one participant. **d** Example of the average across all detected SWRs of one participant and the time-frequency transformation of the average SWR. **e**) Average of all detected SWRs across all participants and the time-frequency transformation of the average SWR. **f** Distribution or inter-ripple intervals across all participants and channels. **g** SWR detection procedure. Top: raw electrophysiological data recorded from one channel. Middle: electrophysiological data filtered to the ripple frequency band (80–180 Hz). Bottom: z-scored envelope of the ripple-band signal with the SWR detection threshold of 3 SD above the mean.

To circumvent these limitations of the retina, we developed the UHV-stimulation, in which high-frequency flickering light is combined with a spatial targeting approach, which shifts the retinal stimulation area. This stimulation is based on the hypothesis that it should be feasible to evoke SSVEP in circumscribed cortical areas by selectively stimulating the retinotopic pathways associated with the respective receptive field. Phase-shifting the stimulation of different receptive fields would then allow evoking SSVEP at the same frequency but with very short time delays between neighboring retinotopic areas. We hypothesize that multiple spatially organized SSVEPs at the same frequency, but with different phases, will be integrated laterally between parallel processing pathways in the primary visual cortex. This would allow the summation or non-linear integration of low-frequency SSVEP optimized to the response properties of the visual system to enhance the power of high-frequency oscillations (Fig. 1b). The summation of the multiple low-frequency SSVEP would then result in a high-frequency SSVEP with a center frequency equal to the number of simultaneous low-frequency SSVEP times their frequency (e.g., 4 * 36 Hz = 144 Hz). A non-linear integration of the low-frequency SSVEP would likely result in a broadband power increase.

### Apparatus

We implemented the UHV-stimulation using PsychoPy 2022.2.5^36^ and designed two different stimulation conditions to selectively and sequentially target neighboring retinal areas with high-frequency flickering light by moving black and white circular retinotopic checkerboard wedges either across the full circle (FC) or across a semi-circle on the bottom half (BH) of the screen (Fig. 1a)^37^. For FC stimuli, the circular stimulus was segmented into twelve 30° sections, and four sections in a cross-shape were presented simultaneously in one screen refresh cycle. In the next screen refresh cycle, the clockwise neighboring segments were activated, which was repeated in the next cycle. Thus, the first segment was activated again after two “off” cycles. For BH stimuli, the bottom half of the circular stimulus was segmented into eight 20° segments, and the two vertically mirrored segments were presented simultaneously in one screen refresh cycle. In the next screen refresh cycle, the lower neighboring segments were presented. Thus, the first segment was activated again after three “off” cycles. Throughout the experiment, participants were asked to fixate on a central fixation cross to ensure the stimulation of the same retinal areas. Due to the “off” cycles, each retinal area was stimulated with a lag of at least 10 ms, and neighboring areas were stimulated at a lag of 8.33 ms or 6.94 ms (i.e., 120 Hz and 144 Hz screen refresh frequency). Thus, each section had a repeat frequency of the screen refresh frequency divided by the number of stimulus sections (e.g.: 144 Hz/4 = 36 Hz). In the baseline recording, participants were shown a static retinotopic checkerboard identical to the pattern used for the FC stimulation to keep low-level visual input identical between baseline and stimulation. Baseline recordings were conducted for three minutes before and after the experiment to avoid biasing the data towards decreased SWR density due to stress before or increased SWR density due to fatigue after the experiment. All other conditions were presented in random order. Stimuli were presented on a 24-inch PC screen (ASUS ROG Strix XG258).

### Participants

Eight participants (*N* = 8, 5 f, 3 m, 21–62 years, self-reported sex and age) suited to take part in the experiment were identified by the attending physicians and informed about the experiment and provided written informed consent to participate in the experiment (Table 1). The experiment was conducted in accordance with the 2008 Declaration of Helsinki and was approved by the ethics committee of the University of Potsdam (Application 43/2023) and Ärztekammer Westfalen-Lippe (Application 2024-024-f-S). The current experiment was not considered a clinical trial, because no health-related interventions were applied. All participants were in the hospital to monitor epileptic activity with implanted electrodes for diagnostic purposes. Because the goal for this monitoring is to record epileptic brain activity, all participants were on reduced doses of antiepileptic medication. All recordings were performed in the patients’ rooms in the respective epilepsy monitoring units of the Epilepsy-Centers Berlin-Brandenburg or Bethel. During the recordings, participants were seated in their hospital beds in a reclined position approximately 1 m in front of the screen, on which the stimulation was shown. For each condition (FC and BH) and each frequency (120 Hz, 144 Hz), they were presented with two blocks of 35 trials of 2 seconds of stimulation and 2 seconds inter-stimulus interval, while we recorded neural activity from depth (sEEG) or foramen ovale (FO) electrodes^38^ implanted in the medial temporal lobe directly in the hippocampus or in close proximity to the hippocampus for diagnostic purposes (Fig. 1c). For exploratory purposes, some participants were also presented with either two blocks of 90 Hz or 180 Hz stimulation of both conditions. Due to the unbalanced number of trials between participants, these data were not included in the current analysis. The experiment had no influence on the placement of the electrodes. During the experiment, participants were asked to keep their eyes open and their gaze on a central fixation cross. Fixation was monitored throughout the experiment, and participants were allowed to take self-paced breaks between stimulation blocks. After the experiment, participants were asked whether they detected differences between the stimulation conditions, whether the stimulation had any negative consequences such as eyestrain or headaches, and whether they experienced seizures during the experiment.

**Table 1:** Participant characteristics including sex, age range, implantation type, side, the target area, the number of included channels, and the site of the experiment

Sex	Age	Type	Side	Area	Chan.	Epilepsy Center
Female	20–25	sEEG	Left	Hippocampus PHG	3 3	Bethel
Female	20–25	FO	Bilateral	Hippocampus	4	Berlin-Brandenburg
Female	30–35	FO	Bilateral	Hippocampus	2	Berlin-Brandenburg
Male	40–45	sEEG FO	Left Bilateral	MTL Hippocampus	8 4	Berlin-Brandenburg
Male	45–50	sEEG	Bilateral	Hippocampus	6	Bethel
Female	35–40	sEEG	Bilateral	Hippocampus	7	Bethel
Male	40–45	FO	Right	Hippocampus	1	Berlin-Brandenburg
Female	60–65	sEEG	Left	PHG	2	Bethel

sEEG indicates depth electrode implantations, FO indicates foramen ovale implantations, PHG indicates parahippocampal gyrus, MTL indicates medial temporal lobe.

### Data processing

For the data processing, the entire stimulation interval for each frequency and condition combination was considered, and SWRs were detected between the onset of the first stimulation trial and the offset of the last stimulation trial. In other words, for each condition, 2 blocks * 35 trials * 4 s = 280 s of data were analyzed. Data were preprocessed using the MATLAB toolbox FieldTrip^39^, including downsampling the data to 1000 Hz and bipolar referencing between neighboring electrodes to create locally independent recording channels^40^. Electrode location was visually screened with the BioImage Suite^41^ where MRI and CT data were available, or from the electrode placement table provided after the implantation, and bipolar channels within or close to the hippocampus were selected (*n* = 51). Next, event-related potentials (ERPs) to the stimulus onset across all conditions and selected channels were computed. To this end, data were filtered with a windowed-sinc FIR 1 Hz highpass and a 45 Hz lowpass filter^42^. Channel-wise ERPs were z-transformed by subtracting the mean across the 500 ms pre-stimulus interval from each sample point and dividing the results by the standard deviation of the 500 ms pre-stimulus interval. Using these z-transformed ERPs, the visually responsive channels (*n* = 42) were identified based on the presence of a peak in the rms-transformed ERP after stimulus onset of z > 2.58 (99.5%). For each channel, the peak amplitude and latency of the rms of the z-scored ERP were collected for further analyses.

Finally, SWRs were detected in the visually responsive channels following established routines^8,43,44^. For this, signals from the visually responsive channels were bandpass filtered between 80 and 180 Hz (windowed-sinc FIR filter) and the instantaneous analytic amplitude was computed using a Hilbert transform. The signal was then squared and smoothed (1-dimensional Gaussian filter with sigma = 4 SD), and the mean and SD were computed across the entire experimental duration to define the threshold for event detection. When recording neural activity from implanted electrodes in epilepsy patients, it is important to distinguish hippocampal ripples from pathological neural activity. To discard data segments comprising epileptic events, events that exceeded 7 SD above the mean were marked as potential artifacts. In line with previous publications, in which signal-detection thresholds between 2 and 4 SD above the mean were chosen^45^, events from the squared original signal that exceeded 3 SD above the mean were selected as candidate SWR events (Fig. 1g). Event duration was expanded until ripple power fell below 2 SD. Candidate SWR events coinciding with artifacts and/or shorter than 15 ms and/or comprising fewer than five peaks were excluded from further analyses. Adjacent SWR events whose onset latency was below 30 ms were merged to one event (see Fig. 1d for an example of the average SWR of one participant and Fig. 1e for the grand average SWR across all participants). From the visually responsive channels (*n* = 42), only those channels in which at least one SWR was detected were included in the statistical analyses (*n* = 40).

### Statistics and reproducibility

For the statistical analysis, we treated each channel (*n* = 40) as an independent observation, and for the comparison between different stimulation frequencies and conditions, only those channels on which SWRs were detected were included. Thus, each channel was measured repeatedly. For each channel, we computed the number of SWRs per second during the baseline and during the stimulation interval of the different conditions and stimulation frequencies. Moreover, we transformed each SWR into time-frequency space using a wavelet analysis (5 cycles per frequency, 80 to 180 Hz, +/−150 ms around the negative SWR peak) and for each SWR computed the z-score of the overall power, z-score of the peak amplitude, peak frequency, and duration. We then used linear models from the “nlme”-package^46^ in R^47^ to compute analyses of variance (Type-III Wald F-tests) to evaluate the hypothesis that the number of SWRs during the stimulation is larger than during the baseline for the factors Frequency (Baseline, 120 Hz, 144 Hz) and Condition (Baseline, FC, BH). In these analyses, we accounted for repeated measures across channels. Because not all levels were repeated across all conditions (e.g., the baseline condition with the stimulation frequency of 0 Hz was not repeated for BH and FC conditions), we first examined the relationship between the number of SWRs and the stimulation frequency (across all conditions) and then examined the relationship between the number of SWRs and the stimulation condition (across all frequencies) in separate analyses. Because the number of SWRs per second is Poisson distributed, values were log-transformed before the analysis. Furthermore, we examined effects of the stimulation on the number of SWRs by post-hoc linear models split by stimulation condition, to examine whether the number of SWRs during either FC or BH stimulation was selectively modulated by one or the other stimulation frequency. Significant effects were followed up by pairwise Holm-corrected independent-samples t-tests using the “emmeans”-package^48^. In the same step, contrast confidence intervals and Cohen’s d as effect size estimates were computed. We carried out similar analyses for the power, amplitude, frequency, and duration of SWRs, as well as for the number of SWRs in the baseline recording before and after the stimulation. Finally, to explore the connection between visual evoked activity and SWR generation, we correlated the log-transformed mean number of SWRs per channel with the log-transformed mean ERP peak amplitude and latency per channel using two-sided, pairwise Pearson’s correlation.

## Results

This exploratory experiment investigates the hippocampal response to UHV-stimulation using intracranial recordings from electrodes implanted in or near the hippocampus of patients suffering from drug-resistant epilepsy. We compare the number of SWRs during the two different stimulus designs (FC and BH) and different stimulation frequencies (120 and 144 Hz) of the UHV-stimulation to the number of SWRs during baseline recordings before and after the stimulation (Table 2).

**Table 2:** Mean and SD of the number of SWRs per second for the different stimulation conditions and frequencies

Condition\Frequency	Baseline Mean	Baseline SD	120 Hz Mean	120 Hz SD	144 Hz Mean	144 Hz SD
BH	0.039	0.039	0.036	0.043	0.061	0.066
FC			0.047	0.054	0.045	0.045

Please note that the baseline condition is not repeated for each condition and frequency.

### Descriptive results

For all analyses, 40 channels across 8 participants are considered. For the comparison between different stimulation frequencies and conditions, only those channels on which SWRs are detected in the examined conditions and frequencies are included. Overall, the average number of SWRs per second (mean = 0.070, SD = 0.093, range 0.005–0.564) is lower than during other human intracranial recording experiments^8,9^, potentially due to suboptimal position of the electrodes. Similarly, the time interval between SWRs is larger compared to other experiments (mean = 11.877, median = 4.792, IQR = 12.611, Fig. 1f). Details on the number of SWRs per second in the different stimulation conditions and frequencies and detailed statistical results can be found in the supplementary table S1.

### Effect of UHV-stimulation on the number of SWRs

**Fig. 2:**
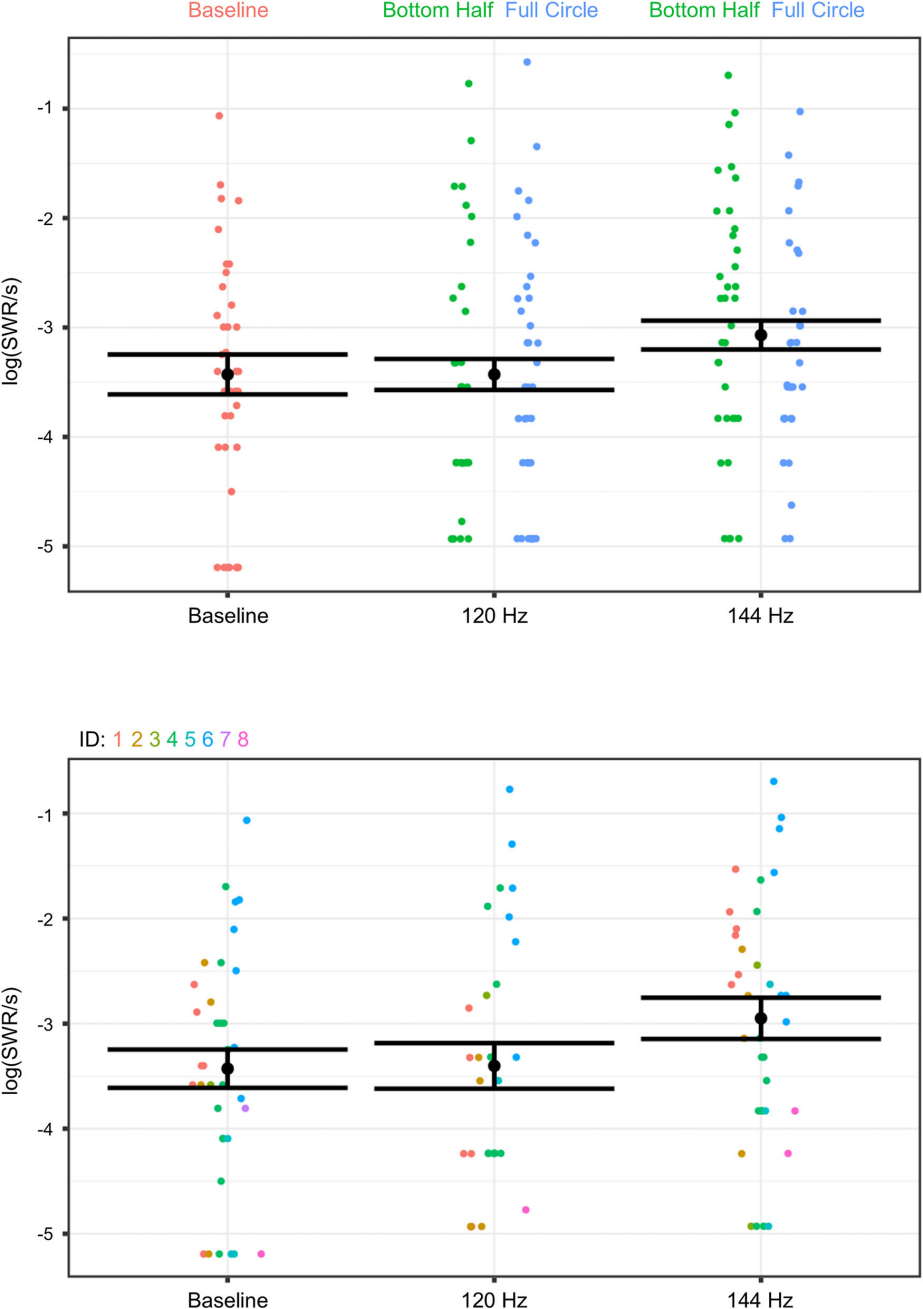
Summary of the log-transformed number of SWRs per second for each channel, condition and participant. Top: Comparison of the number of SWRs between the different stimulation conditions and frequencies. Colors indicate the stimulation conditions (baseline = red, bottom half = green, full circle = blue). Black dots indicate the means, whiskers the SEM. For the BH condition, *n* = 39 channels are considered as biologically independent samples (Baseline = 36 channels, 120 Hz = 30 channels, 144 Hz = 35 channels), for the FC condition, *n* = 40 channels are considered as biologically independent samples (Baseline = 36 channels, 120 Hz = 37 channels, 144 Hz = 32 channels). Bottom: Comparison of the number of SWRs between the different stimulation frequencies for the bottom half stimulation condition. Data are color-coded according to the participants’ ID. Black dots indicate the means, whiskers the SEM.

First, we compare the log-transformed number of SWRs per second between the different stimulation frequencies (Fig. 2 top panel, supplementary table S2) and find a significant effect of the factor Frequency (F(2,56) = 5.1184, *p* = 0.0091), where the number of SWRs is higher during 144 Hz stimulation than during 120 Hz stimulation (t(56) = 3.177, *p* = 0.0073, 95% CI = [0.079, 0.631], Cohen’s d = 0.577, Supplementary Table S3). Second, we compare the number of SWRs between the different stimulation conditions but find no effect of the factor Condition (F(2,57) = 0.7013, *p* = 0.5002, Supplementary Table S4). To explore whether the number of SWRs is selectively modulated by one or the other condition, we split the data by condition and compare the number of SWRs between the different stimulation frequencies in separate post-hoc analyses. For the BH condition (Fig. 2 bottom panel, Supplementary Table S5), a total of 39 channels are considered (Baseline = 36 channels, 120 Hz = 30 channels, 144 Hz = 35 channels), and we find a significant effect of Frequency (F(2,60) = 9.9991, *p* = 0.0002). With unpaired, two-sided t-tests with Holm correction for multiple comparisons (Supplementary Table S6), we find that the number of SWRs is higher during 144 Hz stimulation than during baseline (t(60) = 3.383, *p* = 0.0025, 95% CI = [0.130, 0.826], Cohen’s d = 0.840), and higher than during 120 Hz stimulation (t(60) = 4.209, *p* = 0.0003, 95% CI = [0.257, 0.981], Cohen’s d = 1.088). For the FC condition, a total of 40 channels are considered (Baseline = 36 channels, 120 Hz = 37 channels, 144 Hz = 32 channels), and we find no significant effect of Frequency (F(2,63) = 0.3599, *p* = 0.6992, Supplementary Table S7). To check for changes in the number of SWRs per second due to fatigue over the course of the experiment, we compare the log transformed number of SWRs during the first baseline recording prior to the stimulation to the second baseline after the stimulation with an unpaired, two-sided t-test, and find no difference in the number of SWRs (t(60.961) = 0.0437, *p* = 0.9652, supplementary tables S8 and S9). The analysis of SWR power, amplitude, frequency, and duration indicates no differences between conditions or frequencies (Supplementary Tables S10-S21).

### Correlations between the number of SWRs and ERP amplitude and latency

**Fig. 3:**
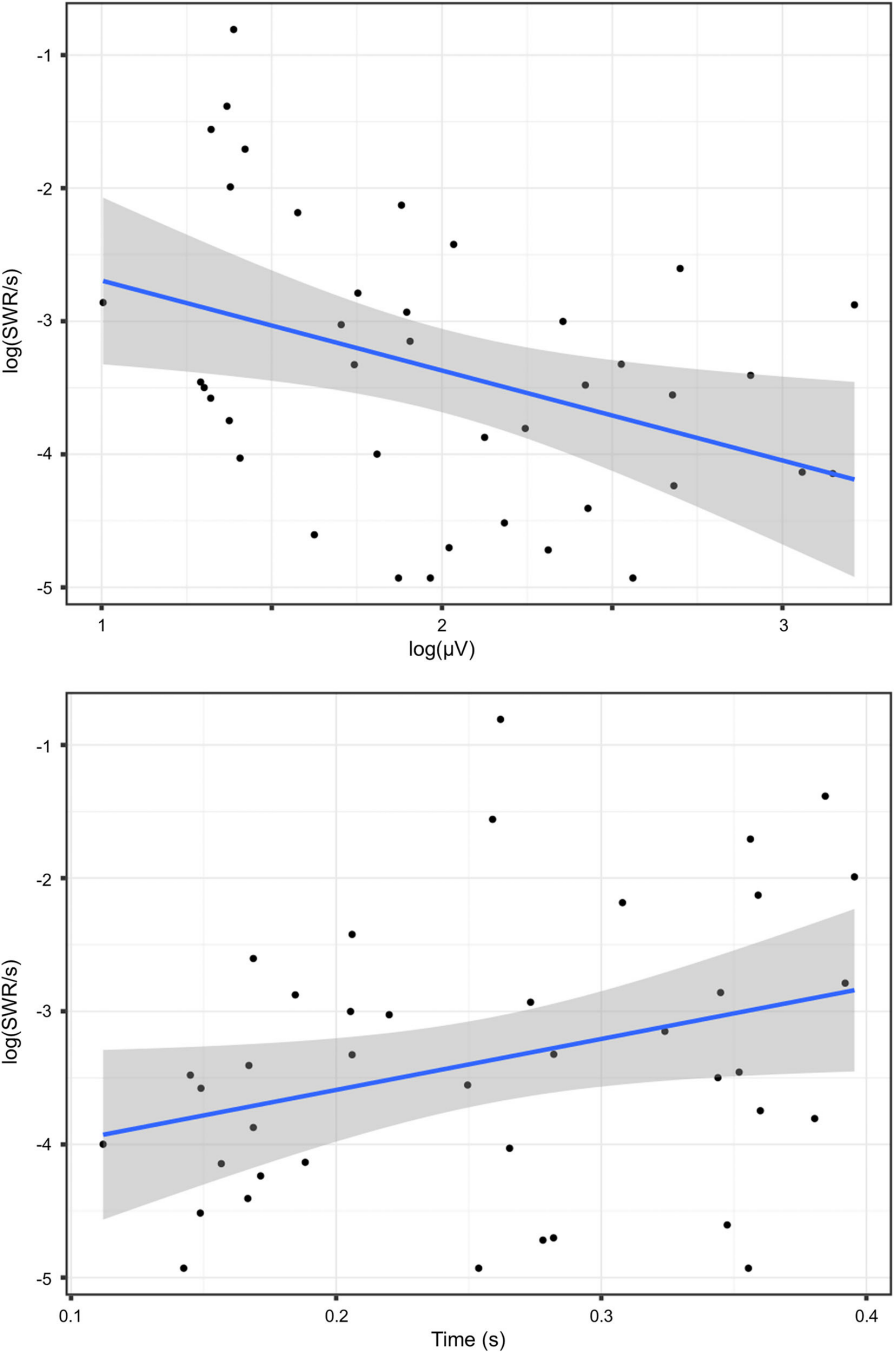
Summary of the relationship between ERP amplitude and latency and the log transformed number of SWRs per second. Top: Correlation between the log-transformed ERP amplitude and log-transformed number of SWR per second. Bottom: Correlation between the ERP peak latency and log-transformed number of SWR per second. Each data point refers to one medial temporal lobe channel. The solid line represents the linear model fit. The shaded area indicates the 95% confidence interval centered on the linear model fit.

Finally, we correlate the average number of SWRs across all conditions and frequencies with the ERP peak amplitude and latency using two-sided, pairwise Pearson’s correlations (Fig. 3). In these analyses, we find a negative correlation between the number of SWRs and the ERP amplitude (r = −0.3765, t(38) = −2.5054, *p* = 0.0166, 95% CI = [−0.615, −0.073]), and a positive correlation with the ERP peak latency (r = 0.3162, t(38) = 2.0551, *p* = 0.0468, 95% CI = [0.005 0.571]). While several channels show early ERP peaks with large amplitudes after around 100 to 200 ms, the channels with the highest number of SWRs show the peak later in time with less prominent peaks.

## Discussion

As we age, memory formation, consolidation, and recall decline. This cognitive decline is amplified in dementia, and despite decades of research, a memory enhancement or therapy for memory loss in dementia remains elusive^49,50^. Sharp-wave ripples play a prominent role in memory^2^, and are impaired in Alzheimer’s Disease, which makes them a prime target for interventions aimed at improving memory in dementia^15^. Electrophysiological recordings showed that ripple oscillations occur in the neocortex and the hippocampus^5^, that they phase-synchronize across long distances, and that neocortical ripples often precede hippocampal ripples during wakefulness^6^. While established methods exist to induce SWRs invasively^19–21^, currently no methods are available for the safe and noninvasive induction of SWRs in humans. As a first step towards this goal, we explored ultra-high frequency flickering light stimulation as a visual stimulation tool. Flickering visual stimulation can lead to steady-state visual evoked potentials, which can influence ongoing brain activity^33^. Thus, it may be possible for ultra-high frequency SSVEP in the ripple frequency range to propagate beyond sensory areas and influence ongoing brain activity in the hippocampus to improve memory. To test this hypothesis, we conducted an exploratory electrophysiological experiment in humans, using ultra-high frequency visual stimulation (UHV-stimulation). We predicted that UHV-stimulation would increase the number of SWRs relative to a resting-state baseline. In line with this prediction, we observed an increase in the number of SWRs during periods of stimulation compared to a resting-state baseline, before and after the stimulation of 56.41%. This preliminary result indicates that UHV-stimulation can be used as a noninvasive tool to influence SWRs, which offers a promising route to improving memory.

Using direct recordings of SWRs from electrodes implanted in or close to the hippocampus in eight epilepsy patients, we found a larger number of SWRs during stimulation compared to baseline. Interestingly, while the SWR density was increased during stimulation, other SWR properties remained unchanged: The overall power, peak amplitude, peak frequency, and duration did not differ between the baseline and stimulation conditions. This indicates that the UHV-stimulation did not induce artificial oscillations but boosted the occurrence of SWRs. It also shows that the hippocampus does not respond with SSVEP, i.e., we do not observe an entrainment of the stimulation frequency despite the strong visual evoked ERP. Thus, the signal recorded from the hippocampus does not seem to be a linear summation of the multiple low-frequency SSVEPs, but rather a non-linear integration of the spatio-temporally organized stimulation. Importantly, the increase in SWR density was not identical across all stimulation frequencies and stimulus patterns but was restricted to the 144 Hz bottom half stimulation. The increased SWR density thus can’t be the result of flickering light stimulation or visual stimulation per se but appears to be related to the pattern and frequency. Currently, the explanation why the 144 Hz bottom half stimulation was more successful in increasing the number of SWRs than other conditions remains speculative. The full circle stimulation potentially led to signal cancellation at the level of the primary visual cortex due to the simultaneous stimulation of opposite circle segments. Such a configuration would induce activity in electrical dipoles in the primary visual cortex with opposite orientation^37^. In contrast, the stimulus design for the bottom half stimulation avoids this signal cancellation. A previous experiment with flickering visual stimulation indicated resonance phenomena at 40 Hz^33^. In the bottom half stimulation condition, the 144 Hz frequency with four stimulation segments has a stimulation frequency of the single segments of 36 Hz. Thus, a stimulation frequency close to the resonance frequency of the visual system might be most effective in inducing ripple oscillations. Future studies should explore this with optimized stimulation patterns and a wider range of stimulation frequencies.

In the interpretation of the current preliminary findings, several limitations must be considered. Overall, the number of SWRs was lower compared to previous studies on human SWRs, which might be due to the applied artifact detection^8,9^ and the relatively short recording interval of only approximately 140 s per stimulation block, and the recording during alert wakefulness. Future studies should put more weight on the correct identification of hippocampal SWRs and their differentiation from high-frequency oscillations. Unfortunately, the placement of the electrodes in the hippocampus could not be verified in all participants, due to a lack of pre- and postoperative CT and MRI images of the electrodes. Recent methodological developments also suggest more stringent identification of SWRs based on spatiotemporal activation patterns or considering fluctuations in the overall noise level^45,51^. However, the former approach requires microwire recordings in addition to depth electrodes, which were not available in the current dataset. Consequently, in the current analyses, we could not distinguish between canonical SWRs and high-frequency oscillations based on the sharp-wave and ripple pattern that can be observed across multiple layers of hippocampal tissue. The influence of noise fluctuations should be most relevant for longer recordings, while the current dataset comprises multiple short recordings. In future studies, the optimal stimulation pattern and frequency need to be explored, in which the stimulation pattern should also be optimized to the visual cortex architecture^37^. In light of recent results showing an increased number of SWR around event boundaries when participants watched movies, more naturalistic stimuli or stimuli with semantic content should be explored^52^. We found a negative correlation between the SWR density and the visual evoked ERP amplitude, and a positive correlation with the ERP peak latency. This could indicate that those channels in which the largest increase in SWR density occurred are further removed from the visual input signals, while those medial temporal channels with a large visual ERP are more closely related to the input structures, e.g., the entorhinal cortex. Whereas we presented a circular checkerboard stimulus similar to the UHV-stimulus during the baseline recording to keep the low-level visual input comparable, future studies should also use sham stimulation with, for example, random spatio-temporal patterns or patterns that are less likely to lead to SSVEP. Furthermore, future studies should also include participants with electrodes implanted in visual cortical areas to elucidate the mechanism connecting UHV-stimuli to cortical and hippocampal ripple activity as well as analyzing the phase differences between different brain regions. Alternatively, studies in rodents or nonhuman primates could employ more targeted electrode implantations to elucidate the signal propagation. In the current experiment, participants were fully aware of the different stimulation conditions and the purpose of the experiment, and the experimenters were aware of the present stimulation condition. During data analysis, the preprocessing and SWR detection were fully automated. Nevertheless, future studies should employ double-blind designs to avoid biases and blind stimulus conditions during data analysis. Future studies should also aim to employ non-invasive recording methods capable of recording signals from deep neural generators such as magnetoencephalography in the non-epileptic population^53,54^. While we employed a conservative approach to differentiate epilepsy-related artifacts from SWRs, it is possible that signal processing in patients with pharmacoresistant mesiotemporal epilepsy differs from physiologically intact neural networks. Whereas all participants were off or on low doses of antiepileptic medication, future studies should furthermore record the medication status, which could help explain the differences in SWR density between participants. The current experiment was conducted in a routine clinical setting, in which the patients receive a reduced dose of antiepileptic medication. Future studies should record the current medication relative to the standard treatment in order to assess the potential effect of some but not all antiepileptic drugs^55,56^.In summary, this exploratory and preliminary experiment indicates that it is possible to use UHV-stimulation to induce SWRs. However, it is still unclear whether this has a positive effect on memory or the cognitive decline in dementia and AD. Future experiments should therefore consider the effect of UHV-stimulation in the context of memory tasks to elucidate whether this increase in SWR density translates to a change in behavior.

Various noninvasive stimulation techniques have emerged as potential treatments for AD^24,57–59^. While some of these methods have demonstrated initial promise, their underlying mechanisms of action remain subject to debate. UHV-stimulation uniquely focuses on the hippocampus and operates through a well-understood mechanism (i.e., SWR induction), distinguishing it from many other less specific interventions: UHV-stimulation appears to significantly increase the number of SWRs compared to baseline. Frequency-modulated visual flicker stimulation has been safely used in humans for more than a century^60^. It has also emerged as perhaps the most effective method of neural entrainment, where the frequency of an external, sensory flicker is synchronized to the brain’s oscillatory activity^33,61^. The risk of triggering an epileptic seizure is a great concern of any form of visual flicker. However, this is mostly a risk for stimulation frequencies between 3 and 70 Hz^62^. In the current experiment, interviews conducted after the experiment did not indicate negative consequences or adverse effects of the stimulation, despite applying the stimulation in a sample of epilepsy patients for approximately one hour, which underscores that the UHV-stimulation is safe for human application, even in vulnerable populations. While the participants in this experiment suffered from focal epilepsy that is rarely associated with photosensitivity^63^, no participant reported seizures associated with stimulation. Therefore, UHV-stimulation is a safe, noninvasive stimulation protocol, which could possibly be applied as AD prevention and therapy with a low treatment burden targeted at the neural mechanisms of memory.

Currently, it is not clear how the neocortical signals propagate to the hippocampus to induce SWRs. A recent fiber-tracking study in humans showed direct anatomical connections between the visual cortex and dorsal hippocampus^64,65^. Similar anatomical connections between occipital and temporal cortical areas have been summarized as the inferior longitudinal fasciculus (ILF), which appears “to mediate the fast transfer of visual signals to anterior temporal regions”^66^. Among many other functions, it has also been implicated in visual memory^67^. Thus, it is conceivable that neocortical activity propagates via these direct anatomical connections to the dorsal hippocampus. Moreover, Pedrosa and colleagues suggested that in mice, bidirectional interactions between retrosplenial cortex and hippocampus mediate information transfer during memory consolidation, in which neocortical activity could bias hippocampal activity^68^. The mechanism underlying this information transfer in distributed neocortical networks could be frequency-specific functional connectivity, in which different frequencies subserve different directions of information transfer^69,70^. Interestingly, the simultaneous occurrence of neocortical and hippocampal ripple oscillations has been hypothesized as one mechanism underlying this information transfer during memory encoding, consolidation and retrieval^6^. During wakefulness, neocortical ripples lead hippocampal ripples and could trigger the occurrence of SWRs. Similarly, UHV-induced neocortical high-frequency activity could induce SWRs.

Due to the low risk profile, UHV-stimulation might be further explored as a preventive measure for people with an identified risk to develop AD or MCI, and for early-stage AD patients. It could also be applied to support memory in normal aging, where a decline of the number of SWRs and cognitive functioning has been observed^71,72^, or in other neurological or psychiatric diseases which exhibit SWR aberrations and impaired cognition, such as epilepsy or schizophrenia^73–75^. Overall, the UHV-stimulation detailed here could be further developed to stimulation with direct beneficial effects on memory and cognitive functioning in normal aging and psychiatric diseases by increasing the number of SWRs.

## Conclusions

Converging evidence from previously published research supports the hypothesis that increasing the number of SWRs is beneficial for memory and the cognitive symptoms of AD. In this exploratory study, we show that UHV-stimulation increases the number of SWRs. Importantly, we observe no unwanted adverse effects or complications of the UHV-stimulation in the vulnerable population included in this experiment. These preliminary findings open the door to exploring UHV-stimulation as a tool to improve memory.

## Supplementary information

Transparent Peer Review file

Supplementary Material

## Data Availability

Processed and anonymized electrophysiological data are available as open data in the Center for Open Science repository https://osf.io/yck7t/. Source data underlying Figs. 1–3 can be accessed from the “Output” folder in https://osf.io/yck7t/files/osfstorage. MRI and CT data can’t be made available due to data protection regulations.

## Code availability

Analysis code for the processing of electrophysiological data and statistical analyses can be found in the Center for Open Science repository 10.17605/OSF.IO/YCK7T. Raw data preprocessing and visualization were performed using the FieldTrip toolbox for MATLAB. SWR detection was performed with custom-written code in Python version 3.10. Statistical analyses were performed with custom-written code in R version 4.5.0.
