# Supplementary material for: High-frequency visual stimulation can increase medial temporal lobe ripple oscillation density
Source: Commun Med (Lond). 2026 Aug 10;6:434. doi: 10.1038/s43856-026-01846-6 (PMC13458760; doi:10.1038/s43856-026-01846-6)
Supplement: Supplementary file 1 [file 43856_2026_1846_MOESM1_ESM.pdf]

nature portfolio

Peer Review File

High-frequency visual stimulation can increase medial temporal lobe ripple oscillation density

Corresponding Author: Dr Julian keil

This file contains all reviewer reports in order by version, followed by all author rebuttals in order by version.

Version 0:

Reviewer comments:

Reviewer #1

(Remarks to the Author)
The study aims to increase hippocampal ripple frequency in humans using ultra-high frequency visual stimulation (UHV-stimulation), employing a newly developed stimulation paradigm. Given the established role of ripple oscillations in memory and cognition, the finding that high-frequency visual stimulation of the neocortex can enhance hippocampal ripple activity is intriguing. The additional observation that this effect exhibits frequency specificity further strengthens the potential significance of the work.
However, the manuscript currently lacks sufficient explanation in several key areas, which makes it challenging for readers to fully understand and evaluate the findings. More detailed descriptions would substantially improve clarity.

Major
1. Regarding SWR detection, the authors state that a conservative detection approach was used because recordings were obtained from epilepsy patients, and large-amplitude events might therefore reflect artifacts (lines 222–224). While this reasoning is understandable, the justification for the chosen detection threshold remains unclear. The authors also note that their thresholding approach differs from previous human intracranial recording studies (lines 270–271). It would be helpful to demonstrate how sensitive the results are to different threshold settings, or at least to provide a more rigorous rationale for the chosen values.
2. The description of the electrode configuration requires clarification. The manuscript mentions 42 channels, but it is unclear whether this refers to 42 channels per participant or 42 channels in total. In some patients, electrodes were implanted bilaterally, the total number of channels would presumably be higher. This raises the possibility that “42 channels” actually refers to 42 data points. However, the numbers of data points in Fig. 2 are fewer than 42. Clearer reporting of the electrode layout and the number of channels per participant is necessary.
3. The sample presentation across stimulus frequencies is difficult to follow. The number of data points and participants decreases markedly at 180 Hz, and it appears that the authors discontinued the 180-Hz condition partway through the study (lines 165–166), thereafter changed with 90-Hz stimulation. Therefore, the post-hoc 144–180 Hz analysis in the Supplementary Materials seems to have limited value. The authors should consider excluding the 180-Hz data entirely and re-running all relevant statistical analyses.
Additionally, the manuscript does not explain why the number of samples differs among participants. Clear reporting of inclusion/exclusion criteria and trial counts is essential for interpretation.
4. Although it is understandable that the baseline condition was not repeated, it would nevertheless be informative to visualize within-condition changes across frequencies (Fig. 2 top) and within-participant changes across frequencies (Fig. 2 bottom). For example, adding figures of plots connecting each data point (spaghetti plot) in Supplementary Material would help readers understand the variability and directionality of changes.

Minor. Ripple events can occur in rapid succession, yet the manuscript does not explain how such consecutive events were identified or handled. Although a 1000-Hz sampling rate is above the Nyquist limit of ripples, it is relatively low for resolving high-frequency ripple components; the authors should comment on whether a higher sampling rate would have improved detection fidelity.
Ripple events can occur in rapid succession, yet the manuscript does not describe how such consecutive events were identified, segmented, or excluded from analysis. Although a 1000-Hz sampling rate is above the Nyquist limit for ripple detection, it remains relatively low for accurately resolving high-frequency ripple components. The authors should comment on whether the use of a higher sampling rate would have improved detection fidelity or altered ripple quantification. In

addition, it is unclear whether the authors distinguished high-frequency oscillations from canonical sharp-wave ripples (SWRs).

Overall, the study presents potentially important findings, but substantial clarification and improved methodological transparency are needed before the results can be fully evaluated.

Reviewer #2

(Remarks to the Author)
This study introduces a non-invasive ultra-high-frequency visual stimulation (UHV-stimulation) paradigm. Using spatially and temporally modulated 144 Hz flicker stimulation increased the density of sharp-wave ripples (SWRs) in hippocampal electrode recordings from patients with epilepsy. The approach is innovative, aiming to establish a correlation between visually evoked ripples and cognitive function and demonstrating potential translational applications in aging and Alzheimer's disease. This work attempts to alter the brain state through non-invasive stimulation. Nonetheless, limitations include a small sample size, absence of robust causal evidence, limited statistical power, and insufficient methodological details.

Major concerns:
1. The authors reference literature indicating that cortical SWRs may exert influence on hippocampal SWRs. Although experimental results suggest that ultra-high-frequency visual stimuli can augment hippocampal SWRs density, they did not concurrently record cortical LFP signals—specifically from the visual cortex—rendering it impossible to elucidate the mechanism connecting ultra-high-frequency visual stimuli to cortical and hippocampal ripple activity. Moreover, the lack of cortical signals prevents further analysis of oscillatory phase differences between the two brain regions.
2. The title mentions flicker visual stimulation can “increase” SWRs in the hippocampus; it accurately reflects the study's findings—namely, that the stimulation “increased” or ‘promoted’ the occurrence of endogenous SWRs, rather than directly “inducing” ripple oscillations in the hippocampus. Based on the results of the most effective 144Hz stimulation, after 70 2-second stimulation trials, the count of hippocampal SWRs was around 13-14, indicating limited effectiveness. Relying solely on the increased count of SWRs within the overall stimulus duration to infer causality and even functional roles of the visual stimuli is insufficient. Since visual stimuli do not directly induce SWRs, the effectiveness of visual stimuli in eliciting SWR increases should be evaluated against a reference standard, and more optimal stimulation methods should be explored. For example, incorporating natural image stimuli as a reference is recommended.
3. The influence of increasing SWRs is only observed in the 144 Hz BH condition, with no significant effects found at other frequencies or within FC conditions. This is an exciting yet underexplored finding. The authors are encouraged to investigate further the reasons behind the particular effectiveness of 144 Hz and the BH mode. This should be a primary focus in the discussion section.
Minor concerns:
1. The configuration and precise positioning of the 42 recording electrode sites are unclear.
2. Figure 1 D and E lack a colorbar.
3. The number of data points per patient varies across each frequency stimulation experiment in Figure 2 bottom, particularly in the 180 Hz stimulation group, which contains only a small number of data points.
4. It is unclear whether full circular images were shown to patients during the baseline recording phase.

Reviewer #3

(Remarks to the Author)
This manuscript presents a method for stimulating the brain with a flickering visual stimulus in the sharp-wave ripple frequency band (80–180 Hz) to test whether hippocampal intracranial electrophysiological recordings show increased sharp-wave ripple incidence during stimulation compared with baseline. The authors hypothesize that increasing sharp-wave ripples—known to support memory formation and recall in animals and humans—through noninvasive high-frequency visual stimulation could ultimately aid in addressing age- and dementia-related memory decline.

While flickering stimulation has previously been used in the gamma range in human studies, typically up to 80 Hz, this study employs stimulation frequencies up to 180 Hz, reaching the upper end of the ripple band and thereby enabling noninvasive stimulation at frequencies associated with memory-related processes. This approach holds promise for both basic and applied research.

The intracranial recordings provide direct evidence for an increase in sharp-wave ripple incidence. However, there are several concerns regarding the study design, data analysis, and interpretation of the results.

Because visual cortical activity was not recorded, it is unclear whether the stimulation effectively engaged cortical areas or whether the visual cortex and hippocampus synchronized in a way that would support efficient information flow. As the participants had temporal lobe epilepsy, potential effects of medication on ripple incidence also cannot be excluded. Moreover, sharp-wave ripples were recorded only during stimulation and not after subsequent sleep, which is known to play a key role in memory-related ripple activity. The stimulation paradigm also lacked spatial features, although ripples are typically associated with spatial information processing. Additionally, sharp-wave ripples are brief (~100 ms) intermittent events occurring seconds apart, whereas this study used many 2-s visual stimulations separated by 2-s intervals and tested

multiple frequencies within each session, making the paradigm quite unlike spontaneous brain activity.

In the analysis, the pre- and post-stimulation 3-minute baselines were combined, which could obscure any stimulation-related changes in subsequent neural activity. Differences between pre- and post-baseline periods should be examined, and if present, the data reanalyzed accordingly.

The protocol is referred to as ultra-high-frequency visual stimulation. Given that the brain exhibits faster ultra-high-frequency oscillations in the 200–600 Hz range, the term “high-frequency visual stimulation” or “ripple-range visual stimulation” may be more appropriate.

The analysis of mean sharp-wave ripple rate revealed significant effects of stimulation frequency (90, 120, 144, and 180 Hz) and stimulus condition (full circle vs. bottom-half). After finding a significant main effect of condition, separate analyses of each condition may not be necessary or justified.

A recent study by Silva et al. (2025, Nature Communications), showing that movie-watching evokes sharp-wave ripples, should be discussed and compared with the present findings. Despite the noted limitations, most are addressed in the manuscript, and the study offers a meaningful contribution to the literature.

Specific comments:

In the abstract, the Background section presents two separate facts without clearly linking them to a hypothesis, yet the Methods section states that the study tests this hypothesis.

Figure 1: Please indicate whether panel B corresponds to the full-circle or bottom-half stimulus.

In Data Processing, specify how the visually responsive channels used in the analysis were distributed across subjects.

In Table 2, the first column should be labeled “Stimulus” or “Condition” rather than “Frequency.”

In the lower part of Figure 2, clarify what “VP” stands for and what the eight differently colored dots represent.

Reviewer #4

(Remarks to the Author)
I co-reviewed this manuscript with one of the reviewers who provided the listed reports. This is part of the Communications Medicine initiative to facilitate training in peer review and to provide appropriate recognition for Early Career Researchers who co-review manuscripts.

Version 1:

Reviewer comments:

Reviewer #1

(Remarks to the Author)
The authors have satisfactorily addressed the concerns I raised. I have no further major concerns.

Reviewer #3

(Remarks to the Author)
The authors have modified the manuscript in accordance with the issues raised by the reviewers.

Minor issues:

Three references are not up to date; details about their web pages are provided below.

Reference 46
https://www.nature.com/articles/s41467-026-68404-5

Reference 52
https://www.nature.com/articles/s41467-025-66562-6

Reference 53
https://www.nature.com/articles/s41467-025-60788-0

Reviewer #4

(Remarks to the Author)
I co-reviewed this manuscript with one of the reviewers who provided the listed reports. This is part of the Communications Medicine initiative to facilitate training in peer review and to provide appropriate recognition for Early Career Researchers who co-review manuscripts.

Open Access This Peer Review File is licensed under a Creative Commons Attribution 4.0 International License, which permits use, sharing, adaptation, distribution and reproduction in any medium or format, as long as you give appropriate credit to the original author(s) and the source, provide a link to the Creative Commons license, and indicate if changes were made.
In cases where reviewers are anonymous, credit should be given to 'Anonymous Referee' and the source.
The images or other third party material in this Peer Review File are included in the article's Creative Commons license, unless indicated otherwise in a credit line to the material. If material is not included in the article's Creative Commons license and your intended use is not permitted by statutory regulation or exceeds the permitted use, you will need to obtain permission directly from the copyright holder.
To view a copy of this license, visit https://creativecommons.org/licenses/by/4.0/

Response to Reviewers

The thank the editor and the reviewers for the critical and supportive review. Line numbers in the response to the reviewers' comments refer to line numbers in the clean version of the revised manuscript.

Referee expertise:

Referee #1: neuroscience, brain anatomy, sharp wave and ripples

Referee #2: neuroscience, learning and memory

Referee #3: cognitive neuroscience, working memory

Referee #4: Early career researcher co-reviewing with one of the Referees

Reviewers' comments:

Reviewer #1 (Remarks to the Author):

The study aims to increase hippocampal ripple frequency in humans using ultra-high frequency visual stimulation (UHV-stimulation), employing a newly developed stimulation paradigm. Given the established role of ripple oscillations in memory and cognition, the finding that high-frequency visual stimulation of the neocortex can enhance hippocampal ripple activity is intriguing. The additional observation that this effect exhibits frequency specificity further strengthens the potential significance of the work.

However, the manuscript currently lacks sufficient explanation in several key areas, which makes it challenging for readers to fully understand and evaluate the findings. More detailed descriptions would substantially improve clarity.

We thank the reviewer for the clear and constructive feedback, that helped us identifying several shortcomings in the analysis and reporting of the data.

Major

1. Regarding SWR detection, the authors state that a conservative detection approach was used because recordings were obtained from epilepsy patients, and large-amplitude events might therefore reflect artifacts (lines 222–224). While this reasoning is understandable, the justification for the chosen detection threshold remains unclear. The authors also note that their thresholding approach differs from previous human intracranial recording studies (lines 270–271). It would be helpful to demonstrate how sensitive the results are to different threshold settings, or at least to provide a more rigorous rationale for the chosen values.

We thank the reviewer for the suggestion to include additional explanations for the chosen threshold values. Previous human intracranial recording studies used SWR detection thresholds between 2 and 4 SD above the mean, as summarized in a recent paper on optimized SWR detection (Van Schalkwijk & Helfrich, 2026). The threshold value of 3 SD adopted in the current data analysis falls into this range. We have extended the explanation of our threshold selection to the methods section (see lines 212ff), and added an illustration of raw data, filtered data and the selection threshold relative to the z-scored data envelope to further demonstrate the sensitivity of the chosen selection threshold (see figure 1G). With respect to optimized SWR detection in general, we have added a paragraph to the discussion section with suggestions for future research (see lines 384ff). Please also note that we identified an error during the review of the SWR detection approach: The step to identify candidate SWR events with less than 30 ms spacing was missing from the previous data analysis. This has been corrected in the current analyses (line 228f), which reduced the overall number of detected SWRs but had no effect on the general results.

2. The description of the electrode configuration requires clarification. The manuscript mentions 42 channels, but it is unclear whether this refers to 42 channels per participant or 42 channels in total. In some patients, electrodes were implanted bilaterally, the total number of channels would presumably be higher. This raises the possibility that “42 channels” actually refers to 42 data points. However, the numbers of data points in Fig. 2 are fewer than 42. Clearer reporting of the electrode layout and the number of channels per participant is necessary.

We agree that the descriptions of the channel selection lacked transparency. The reviewer is correct to assume that channels refer to data points. However, it is important to note that not all channels (n=40) were included in all analyses, which explains the different number of data points between analyses. To alleviate this confusion, we have added further information on the implantation area and number of channels per area and participant to table 1. Furthermore, we have added the number of channels included in the different analyses in the text.

3. The sample presentation across stimulus frequencies is difficult to follow. The number of data points and participants decreases markedly at 180 Hz, and it appears that the authors discontinued the 180-Hz condition partway through the study (lines 165–166), thereafter changed with 90-Hz stimulation. Therefore, the post-hoc 144–180 Hz analysis in the Supplementary Materials seems to have limited value. The authors should consider excluding the 180-Hz data entirely and re-running all relevant statistical analyses.
Additionally, the manuscript does not explain why the number of samples differs among participants. Clear reporting of inclusion/exclusion criteria and trial counts is essential for interpretation.

As before, we agree that the description of the stimulation procedure lacked transparency. Furthermore, we agree that including stimulation frequencies only applied in some, but not all participants is problematic. Therefore, we now only focus the data analysis on the baseline, 120 Hz and 144 Hz stimulation frequencies in bottom half and full circle stimulation conditions, which were applied in all participants. With respect to the number of samples included for each participant, we have added the number of channels per participant to table 1. Please note that in all analyses, we consider the channel per participant as the unit of observation and considered the whole stimulation interval for each frequency and condition combination as one trial. Therefore, in the current analyses, the trial count is identical for each channel. Because we excluded channels without SWRs in each analysis, the number of channels included for each analysis differs depending on whether an SWR was detected in a channel in a frequency and condition combination. We have extended the description of the stimulation in the methods section accordingly (see lines 175ff, 192ff, and 233ff).

4. Although it is understandable that the baseline condition was not repeated, it would nevertheless be informative to visualize within-condition changes across frequencies (Fig. 2 top) and within-participant changes across frequencies (Fig. 2 bottom). For example, adding figures of plots connecting each data point (spaghetti plot) in Supplementary Material would help readers understand the variability and directionality of changes.

Whereas we agree that adding connecting lines between the data points across conditions could illustrate the change across conditions, this is not feasible for the current data analysis. As described above, we considered each channel as the unit of observation, and only included those channels, that showed at least one SWR in a given frequency and condition combination. For example, a channel that showed SWRs in the baseline, but not in the 144 Hz bottom wedge condition would only be included in the estimation of SWR density of the former, but not the latter condition. This results in a different number of channels for each condition, and an overall unpaired design. We now report the number of channels for each condition in the results section.

Minor. Ripple events can occur in rapid succession, yet the manuscript does not describe how such consecutive events were identified, segmented, or excluded from analysis. Although a 1000-Hz sampling rate is above the Nyquist limit for ripple detection, it remains relatively low for accurately resolving high-frequency ripple components. The authors should comment on whether the use of a higher sampling rate would have improved detection fidelity or altered ripple quantification. In addition, it is unclear whether the authors distinguished high-frequency oscillations from canonical sharp-wave ripples (SWRs).

We thank the reviewer for pointing out this omission. As mentioned above, we in fact missed the treatment of consecutive events with short temporal spacing. We have added the missing information to the methods section (lines 228f). With respect to the ripple quantification and the discrimination of canonical SWRs from high-frequency oscillations, we have extended the discussion of limitations (see lines 380ff). In short, due to the lack of recording channels across multiple layers of hippocampal tissue, we can't identify the typical sharp-wave and ripple pattern seen in rodent recordings. Due to this lack of ability to clearly delineate canonical hippocampal SWRs, we only mention "medial temporal lobe ripple oscillations" instead of SWRs in the title. Despite these limitations, we would argue that a 1000 Hz sampling rate should be sufficient to resolve oscillations in the ripple band (80-180 Hz), because the Nyquist frequency (500 Hz) is more than twice the high-pass filter frequency.

Overall, the study presents potentially important findings, but substantial clarification and improved methodological transparency are needed before the results can be fully evaluated.

We thank the reviewer for the constructive feedback.

Reviewer #2 (Remarks to the Author):

This study introduces a non-invasive ultra-high-frequency visual stimulation (UHV-stimulation) paradigm. Using spatially and temporally modulated 144 Hz flicker stimulation increased the density of sharp-wave ripples (SWRs) in hippocampal electrode recordings from patients with epilepsy. The approach is innovative, aiming to establish a correlation between visually evoked ripples and cognitive function and demonstrating potential translational applications in aging and Alzheimer's disease. This work attempts to alter the brain state through non-invasive stimulation. Nonetheless, limitations include a small sample size, absence of robust causal evidence, limited statistical power, and insufficient methodological details.

We thank the reviewer for the critical evaluation of our work. According to the feedback, we have amended the discussion to clearly highlight the strengths and limitations of the current experiment.

Major concerns:

1. The authors reference literature indicating that cortical SWRs may exert influence on hippocampal SWRs. Although experimental results suggest that ultra-high-frequency visual stimuli can augment hippocampal SWRs density, they did not concurrently record cortical LFP signals—specifically from the visual cortex—rendering it impossible to elucidate the mechanism connecting ultra-high-frequency visual stimuli to cortical and hippocampal ripple activity. Moreover, the lack of cortical

signals prevents further analysis of oscillatory phase differences between the two brain regions.

We agree with the reviewer that the current experiment has limitations with respect to elucidating the connection between ultra-high frequency visual stimulation, cortical activation and hippocampal SWR density. Unfortunately, we don't have any influence over the placement of the intracranial electrodes in the human participants. We have added these suggestions for future experiments to the discussion (see lines 652ff). However, we're currently running electrophysiological experiments in mice to elucidate the effects of the stimulation in the visual cortex (a preprint can be found here: https://www.biorxiv.org/content/10.1101/2025.01.14.632930v1) and in the hippocampus (in preparation). Unfortunately, we're not allowed to cite manuscripts under review in Nature Communications Medicine.

2. The title mentions flicker visual stimulation can "increase" SWRs in the hippocampus; it accurately reflects the study's findings—namely, that the stimulation "increased" or 'promoted' the occurrence of endogenous SWRs, rather than directly "inducing" ripple oscillations in the hippocampus. Based on the results of the most effective 144Hz stimulation, after 70 2-second stimulation trials, the count of hippocampal SWRs was around 13-14, indicating limited effectiveness. Relying solely on the increased count of SWRs within the overall stimulus duration to infer causality and even functional roles of the visual stimuli is insufficient. Since visual stimuli do not directly induce SWRs, the effectiveness of visual stimuli in eliciting SWR increases should be evaluated against a reference standard, and more optimal stimulation methods should be explored. For example, incorporating natural image stimuli as a reference is recommended.

We're thankful for this suggestion on future experiments and have added this suggestion to the discussion section (see lines 393ff). With respect to the optimal stimulation method, we ran two EEG experiments in healthy participants to see which visual stimulation pattern would elicit the strongest visual cortex response, and the manuscripts are currently under preparation. We have already tried to implement a flickering natural image stimulation, but this was not effective in inducing SSVEPs.

3. The influence of increasing SWRs is only observed in the 144 Hz BH condition, with no significant effects found at other frequencies or within FC conditions. This is an exciting yet underexplored finding. The authors are encouraged to investigate further the reasons behind the particular effectiveness of 144 Hz and the BH mode. This should be a primary focus in the discussion section.

At the moment, any discussion of the reasons for the observed effect remains speculative. Nonetheless, we can argue that the full circle condition was probably not effective due to signal cancellation at the level of the primary visual cortex. In contrast, the bottom half stimulation at 144 Hz has a stimulation frequency of 36 Hz

for each of the four stimulus segments. In a previous visual stimulation experiment, a resonant response of the visual system was observed at 40 Hz, so a stimulation close to this frequency might be most effective to induce neural oscillations in the visual cortex. We have added this to the discussion section in lines 363ff.

Minor concerns:
1. The configuration and precise positioning of the 42 recording electrode sites are unclear.

We apologize for this lack of transparency, which was also pointed out by reviewer #1. Accordingly, we have extended table 1 to include more information on the position of the included recording channels.

2. Figure 1 D and E lack a colorbar.

We have added the colorbars.

3. The number of data points per patient varies across each frequency stimulation experiment in Figure 2 bottom, particularly in the 180 Hz stimulation group, which contains only a small number of data points.

We apologize for this lack of transparency. To amend this, we have extended the description of the data processing in the methods section (see lines 175ff, 192ff, and 233ff). Please note that in line with a suggestion by reviewer #1, we have removed the stimulation conditions that were not used in all participants, i.e., we now only report on the baseline, 120 Hz and 144 Hz stimulation frequencies.

4. It is unclear whether full circular images were shown to patients during the baseline recording phase.

We're sorry that we missed including this information, and have extended the description of the baseline condition in line 153ff. In short, during the baseline the participants saw the same pattern as during the full circle stimulation, with the difference that in the baseline, this was just a static image without any flickering.

Reviewer #3 (Remarks to the Author):

This manuscript presents a method for stimulating the brain with a flickering visual stimulus in the sharp-wave ripple frequency band (80–180 Hz) to test whether hippocampal intracranial electrophysiological recordings show increased sharp-wave ripple incidence during stimulation compared with baseline. The authors hypothesize that increasing sharp-wave ripples—known to support memory formation and recall in animals and humans—through noninvasive high-frequency visual stimulation could

ultimately aid in addressing age- and dementia-related memory decline.

While flickering stimulation has previously been used in the gamma range in human studies, typically up to 80 Hz, this study employs stimulation frequencies up to 180 Hz, reaching the upper end of the ripple band and thereby enabling noninvasive stimulation at frequencies associated with memory-related processes. This approach holds promise for both basic and applied research.

The intracranial recordings provide direct evidence for an increase in sharp-wave ripple incidence. However, there are several concerns regarding the study design, data analysis, and interpretation of the results.

We thank the reviewer for the critical review of our work and the valuable suggestions.

Because visual cortical activity was not recorded, it is unclear whether the stimulation effectively engaged cortical areas or whether the visual cortex and hippocampus synchronized in a way that would support efficient information flow.

We agree that the lack of visual cortex recordings renders it impossible to elucidate the effect of the stimulation on the visual cortex and the synchronization between visual cortical areas and the hippocampus. In line with this comment and also the comments by reviewer #2, we have extended the discussion section to highlight this limitation (lines 407ff). Furthermore, we're currently running electrophysiological experiments in mice to elucidate the effects of the stimulation in the visual cortex (a preprint can be found here: https://www.biorxiv.org/content/10.1101/2025.01.14.632930v1) and in the hippocampus (in preparation). Unfortunately, we're not allowed to cite manuscripts under review in Nature Communications Medicine.

As the participants had temporal lobe epilepsy, potential effects of medication on ripple incidence also cannot be excluded.

We apologize for the lack of information on medication. As we now describe in the methods section, all participants were in the hospital to monitor epileptic activity with implanted electrodes for diagnostic purposes. Because the goal for this monitoring is to record epileptic brain activity, all participants were on reduced doses of their usual antiepileptic medication (see lines 170ff). Effects of rapid drug withdrawal cannot be excluded. However, this is unavoidable in human intracranial electrophysiology studies, which are conducted in a routine clinical setting. We have added the lack of detailed information on medication to the discussion section as a suggestion for future research (see lines 425ff).

Moreover, sharp-wave ripples were recorded only during stimulation and not after subsequent sleep, which is known to play a key role in memory-related ripple activity. The stimulation paradigm also lacked spatial features, although ripples are typically associated with spatial information processing. Additionally, sharp-wave ripples are brief (~100 ms) intermittent events occurring seconds apart, whereas this study used many 2-s visual stimulations separated by 2-s intervals and tested multiple frequencies within each session, making the paradigm quite unlike spontaneous brain activity.

We agree that most SWR research indicates increased SWR density during slow-wave sleep, and after spatial learning tasks, especially in rodents. And indeed, our stimulation of 2 s intervals of stimulation is quite unlike spontaneous brain activity. However, the goal of this experiment was specifically to examine whether UHV-stimulation would modulate SWR density during wakefulness and outside of the context of learning experiments. In other words, we consciously abstracted from the usual SWR and memory experiments to focus only on the effect of stimulation. Please note that we compare the SWR density to a baseline condition with constant visual input. If UHV stimulation was detrimental to SWR occurrence, we would see lower SWR density during the UHV blocks. In contrast, we see either equal or higher SWR density during the stimulation blocks compared to the baseline blocks. Future experiments should consider this effect in the context of memory tasks to elucidate whether this increase in SWR density translates to a change in behavior. We have extended the discussion section to include in this in lines 376ff.

In the analysis, the pre- and post-stimulation 3-minute baselines were combined, which could obscure any stimulation-related changes in subsequent neural activity. Differences between pre- and post-baseline periods should be examined, and if present, the data reanalyzed accordingly.

We thank the reviewer for this suggestion and have compared the SWR density between the baseline recordings before and after the stimulation sessions. As we report now in the results section, there was no difference in the number of SWRs between both recording intervals (see line 306ff).

The protocol is referred to as ultra-high-frequency visual stimulation. Given that the brain exhibits faster ultra-high-frequency oscillations in the 200–600 Hz range, the term “high-frequency visual stimulation” or “ripple-range visual stimulation” may be more appropriate.

We use the term “ultra-high” to differentiate the current stimulation from visual stimulation paradigms that use stimulation in the gamma frequency range (e.g., 40 Hz), which could already be considered “high frequency”. Moreover, we have already used this term in a previous publication on the hypothesis of visual brain stimulation to induce SWRs (Keil et al., 2023), we prefer to maintain the wording.

The analysis of mean sharp-wave ripple rate revealed significant effects of stimulation frequency (90, 120, 144, and 180 Hz) and stimulus condition (full circle vs. bottom-half). After finding a significant main effect of condition, separate analyses of each condition may not be necessary or justified.

We agree that further exploration of the stimulation effect split by stimulation conditions is not strictly necessary. However, due to the design of the current experiment, it was not possible to include all factors (Frequency and Condition) in one model without considering the baseline interval multiple times. Thus, the split by frequency should be considered a post-hoc analysis to the first analysis. We have extended the description of the statistical analyses in line 246ff and of the results in line 292ff.

A recent study by Silva et al. (2025, Nature Communications), showing that movie-watching evokes sharp-wave ripples, should be discussed and compared with the present findings.

We thank the reviewer for this suggestion and have included it in the suggestions for future research (see line 395ff).

Despite the noted limitations, most are addressed in the manuscript, and the study offers a meaningful contribution to the literature.

We thank the reviewer for the overall positive feedback.

Specific comments:

In the abstract, the Background section presents two separate facts without clearly linking them to a hypothesis, yet the Methods section states that the study tests this hypothesis.

We thank the reviewer for pointing out this lack of consistency and have formulated the tested hypothesis more clearly in the abstract (see line 32ff).

Figure 1: Please indicate whether panel B corresponds to the full-circle or bottom-half stimulus.

We apologize for this omission. Panel B corresponds to the 144 Hz bottom half stimulation. We have added this information to the figure legend.

In Data Processing, specify how the visually responsive channels used in the analysis were distributed across subjects.

As suggested, we have added this information to table 1.

In Table 2, the first column should be labeled “Stimulus” or “Condition” rather than “Frequency.”

In line with the suggestion, we have diagonally split the top left field of table 2 and labeled it with “Frequency” and “Condition”.

In the lower part of Figure 2, clarify what “VP” stands for and what the eight differently colored dots represent.

“VP” stands for the German word “Versuchsperson”, which means “participant”. We have changed the figure legend to indicate that the color refers to different participants.

Reviewer #4 (Remarks to the Author):

I co-reviewed this manuscript with one of the reviewers who provided the listed reports. This is part of the Communications Medicine initiative to facilitate training in peer review and to provide appropriate recognition for Early Career Researchers who co-review manuscripts.

We welcome the inclusion of early career researchers in the manuscript evaluation process, and are thankful for the critical and constructive remarks.
