# Supplementary material for: High-frequency visual stimulation can increase medial temporal lobe ripple oscillation density
Source: Commun Med (Lond). 2026 Aug 10;6:434. doi: 10.1038/s43856-026-01846-6 (PMC13458760; doi:10.1038/s43856-026-01846-6)
Supplement: Supplementary file 2 [file 43856_2026_1846_MOESM2_ESM.pdf]

Supplementary Material

Number of SWRs per second

All Conditions

	Baseline Mean	Baseline SD	120 Hz Mean	120 Hz SD	144 Hz Mean	144 Hz SD
BH	0.056	0.067	0.069	0.096	0.095	0.110
FC			0.064	0.101	0.067	0.078

Supplementary table S1: Mean and standard deviation of number of SWRs per second for bottom half and full circle conditions per frequency.

Split by Frequency

	num DF	den DF	F-value	p-value
Intercept	1	109	1107.4046	<.0001
Frequency	2	56	5.1184	0.0091

Supplementary table S2: Type-III Wald F-tests, one-sided.

Post-Hoc Analyses

Contrast	Estimate	SE	df	t-ratio	p-value	CI	d
0 – 120	0.0936	0.229	56	0.409	0.6843	-0.472 0.659	0.152
0 – 144	-0.2614	0.229	56	-1.141	0.5177	-0.827 0.304	-0.425
**120 – 144**	**-0.3550**	**0.112**	**56**	**-3.177**	**0.0073**	**-0.631 -0.079**	**-0.577**

Supplementary table S3: Unpaired, two-sided t-tests with Holm correction for multiple comparisons. CI represents the 95% confidence interval, d refers to Cohen's d.

Split by Condition

	num DF	den DF	F-value	p-value
Intercept	1	108	421.6433	<.0001
Condition	2	57	0.7013	0.5002

Supplementary table S4: Type-III Wald F-tests, one-sided.

Only BH stimulation

Split by Frequency

	num DF	den DF	F-value	p-value
Intercept	1	60	373.7208	<.0001
**Frequency**	**2**	**60**	**9.9991**	**0.0002**

Supplementary table S5: Type-III Wald F-tests, one-sided.

Post-Hoc Analyses

Contrast	Estimate	SE	df	t-ratio	p-value	CI	d
0 – 120	0.141	0.145	60	0.971	0.3352	-0.217 0.500	0.248
**0 – 144**	**-0.478**	**0.141**	**60**	**-3.383**	**0.0025**	**-0.826 -0.130**	**-0.840**
**120 – 144**	**-0.619**	**0.147**	**60**	**-4.209**	**0.0003**	**-0.981 -0.257**	**-1.088**

Supplementary table S6: Unpaired, two-sided t-tests with Holm correction for multiple comparisons. CI represents the 95% confidence interval, d refers to Cohen's d.

Only FC stimulation

Split by Frequency

	num DF	den DF	F-value	p-value
Intercept	1	63	446.5412	<.0001
Frequency	2	63	0.3599	0.6992

Supplementary table S7: Type-III Wald F-tests, one-sided.

Only baseline condition

Split by Block: Before vs. after the stimulation

	Before Mean	Before SD	After Mean	After SD
Baseline	0.062	0.069	0.062	0.070

Supplementary table S8: Mean and standard deviation of number of SWRs per second for in the baseline conditions.

	DF	t-value	p-value
Frequency	60.961	0.0437	0.9652

Supplementary table S9: Type-III Wald F-tests, one-sided.

Frequency of SWRs

All Conditions

	Baseline Mean	Baseline SD	120 Hz Mean	120 Hz SD	144 Hz Mean	144 Hz SD
BH	101.213	15.950	98.556	11.791	99.279	12.433
FC			104.330	17.688	98.846	14.997

Supplementary table S10: Mean and standard deviation of SWR frequency for bottom half and full circle conditions per frequency.

Split by Frequency

	num DF	den DF	F-value	p-value
Intercept	1	72	2487.7619	<.0001
Frequency	2	56	0.2885	0.7505

Supplementary table S11: Type-III Wald F-tests, one-sided.

Split by Condition

	num DF	den DF	F-value	p-value
Intercept	1	71	2523.4200	<.0001
Condition	2	57	0.1425	0.8675

Supplementary table S12: Type-III Wald F-tests, one-sided.

Power of SWRs

All Conditions

	Baseline Mean	Baseline SD	120 Hz Mean	120 Hz SD	144 Hz Mean	144 Hz SD
BH	0.502	2.024	0.139	0.782	0.371	0.846
FC			0.537	1.984	0.165	0.602

Supplementary table S13: Mean and standard deviation of SWR power for bottom half and full circle conditions per frequency.

Split by Frequency

	num DF	den DF	F-value	p-value
Intercept	1	72	3.2958	0.0736
Frequency	2	56	0.3070	0.7369

Supplementary table S14: Type-III Wald F-tests, one-sided.

Split by Condition

	num DF	den DF	F-value	p-value
Intercept	1	71	3.3002	0.0735
Condition	2	57	0.0450	0.9560

Supplementary table S15: Type-III Wald F-tests, one-sided.

Amplitude of SWRs

All Conditions

	Baseline Mean	Baseline SD	120 Hz Mean	120 Hz SD	144 Hz Mean	144 Hz SD
BH	0.765	2.543	0.348	1.161	0.933	2.448
FC			0.575	2.014	0.430	1.568

Supplementary table S16: Mean and standard deviation of SWR amplitude for bottom half and full circle conditions per frequency.

Split by Frequency

	num DF	den DF	F-value	p-value
Intercept	1	72	5.5469	0.0212
Frequency	2	56	1.1379	0.2602

Supplementary table S17: Type-III Wald F-tests, one-sided.

Split by Condition

	num DF	den DF	F-value	p-value
Intercept	1	71	5.5279	0.0215
Condition	2	57	0.3056	0.7379

Supplementary table S18: Type-III Wald F-tests, one-sided.

Duration of SWRs

All Conditions

	Baseline Mean	Baseline SD	120 Hz Mean	120 Hz SD	144 Hz Mean	144 Hz SD
BH	0.021	0.006	0.209	0.005	0.021	0.004
FC			0.211	0.006	0.021	0.003

Supplementary table S19: Mean and standard deviation of SWR duration for bottom half and full circle conditions per frequency.

Split by Frequency

	num DF	den DF	F-value	p-value
Intercept	1	72	1896.6402	<.0001
Frequency	2	56	0.1293	0.8790

Supplementary table S20: Type-III Wald F-tests, one-sided.

Split by Condition

	num DF	den DF	F-value	p-value
Intercept	1	71	1859.3271	<.0001
Condition	2	57	0.0911	0.9130

Supplementary table S21: Type-III Wald F-tests, one-sided.
